# Characterization of a Cytokine-Independent STAT5 Activator

**DOI:** 10.3390/biomedicines14051097

**Published:** 2026-05-13

**Authors:** Grace A. Aleck, Yena Jin, Zehui Gu, Adam H. Courtney

**Affiliations:** 1 Department of Pharmacology, University of Michigan, Ann Arbor, MI 48109, USA; aleckg@umich.edu (G.A.A.); zehuigu@umich.edu (Z.G.); 2 Cellular & Molecular Biology Training Program, University of Michigan, Ann Arbor, MI 48109, USA; 3 Rogel Cancer Center, University of Michigan, Ann Arbor, MI 48109, USA

**Keywords:** STAT5, LCK, kinase, cytokine, T cell signaling, CAR-T, NK cell, T cell

## Abstract

**Background**: Cytokine-induced JAK–STAT signaling becomes dysregulated in chronic human diseases, including cancer and autoimmunity, and contributes to immune cell dysfunction. A cytokine-independent approach to activating STAT proteins could “hardwire” pro-survival and effector programs in immune cells to sustain function within diseased tissues. Engineered variants of the herpesvirus saimiri tyrosine kinase interacting protein (TIP) can recruit the SRC family kinase (SFK) LCK to drive STAT phosphorylation and activation. Here, we evaluated the interactome of a TIP-derived, cytokine-independent STAT5 activator and determined whether it could induce STAT5 activation in immune cell lines and primary human CD8+ T cells. **Methods**: A STAT5 activator (aSTAT5) was characterized by proteomics using affinity purification mass spectrometry (AP-MS) to define its interactome and STAT5 binding specificity. STAT5 phosphorylation was assessed in hematopoietic cell lines and primary human CD8+ T cells. **Results**: Proteomic analysis confirmed preferential association of aSTAT5 with STAT5 relative to other proteins. In cell-based assays, aSTAT5 induced robust STAT5 phosphorylation in LCK-expressing NK-92 and Jurkat T cells, whereas phosphorylation was not observed in Raji B cells or RAW 264.7 macrophages despite expression of closely related SFKs and STAT5. Cytokine-independent STAT5 phosphorylation supported the viability of NK-92 cells and primary human CD8+ T cells during cytokine withdrawal and preserved the cytotoxic function of CAR T cells. **Conclusions**: We defined the interactome of a cytokine-independent STAT5 activator and demonstrated its capacity to maintain survival and function in human CD8+ T cells and NK-92 cells. These findings underscore the translational potential of engineered, cytokine-independent STAT5 activation for immune cell therapies.

## 1. Introduction

The herpesvirus saimiri (HVS) infects T cells and uses viral proteins that interface with host factors to co-opt their cellular machinery [1,2]. The HVS tyrosine kinase interacting protein (TIP) recruits the SRC family kinase (SFK) LCK to non-canonical substrates such as STAT proteins to induce the phosphorylation of their regulatory tyrosine residues and activate them. We determined that TIP could be engineered to serve as a platform to induce the phosphorylation and activation of targeted STAT proteins in T cells [3]. This was accomplished by removing portions of the viral protein—specifically the N-terminal region, the transmembrane domain, and an adjacent amphipathic helical region—elements implicated in trafficking and in interactions with other host factors [4,5,6]. The LCK binding motifs of TIP that cause increased kinase activity were retained, as were the adjacent STAT binding sites. These STAT binding sites contain short peptide motifs that are bound by STATs via their SH2 domain when a tyrosine residue within the motif is phosphorylated. It was determined that installation of peptide motifs derived from endogenous cytokine receptors to generate TIP variants enabled specific STAT proteins to be recruited to LCK. A TIP variant that contained a binding site derived from the IL-2 receptor β-chain (IL-2Rβ) could activate STAT5 and maintain CD8+ T cell survival ex vivo in the absence of pro-survival cytokines like IL-2. The effects of the IL-2Rβ TIP variant were determined to be JAK-independent by small molecule inhibition of LCK, which, in the absence of IL-2, blocked STAT5 phosphorylation and prevented T cell viability. When STAT5 was activated in tumor-specific T cells, it was found to promote their persistence and prevent hallmarks of T cell exhaustion in mouse tumor models [3]. This provides evidence that a cytokine-independent approach to activating STAT proteins through aSTAT5 could “hardwire” pro-survival and effector signals in immune cells to maintain their function in diseased tissues, ultimately addressing the dysregulated JAK-STAT signaling often found in chronic human disease. Because cytokine-independent STAT5 activation altered the functional and phenotypic properties of T cells, there is a need to define the protein interaction partners of engineered TIP variants and to determine whether STAT5 can be similarly activated in human hematopoietic cell types that possess closely related SFKs for therapeutic applications.

B and T lymphocytes, as well as myeloid cells and other hematopoietic cells, utilize SFKs to initiate signaling downstream of cell surface receptors. These include antigen receptors such as the T cell receptor (TCR) and B cell receptor (BCR), as well as Fc receptors (FcR). SFKs phosphorylate immunoreceptor tyrosine-based activation motifs (ITAMs) within these receptor complexes, which create a docking site for signaling effectors such as SYK family kinases like ZAP-70. In B cells, LYN phosphorylates ITAMs within the BCR-associated Igα/β chains, whereas ITAMs within the FcγRI-associated γ-chain expressed by macrophages are phosphorylated by HCK or LYN, enabling antibody-dependent cellular cytotoxicity and phagocytosis [7]. SFK function in receptor signaling is coupled to their membrane localization and conformational regulation. SFKs are anchored to the plasma membrane by lipidation of their N-terminus, which positions them near receptor-associated ITAMs. The catalytic kinase activity of SFKs is controlled by phosphorylation of two conserved tyrosine residues located in the C-terminal tail and activation loop [8,9]. The kinase CSK inhibits SFKs through phosphorylation of their C-terminal tail to stabilize their closed autoinhibited conformation. Specifically, the SFK SH2 domain binds the phosphorylated C-terminal tail while the linker between the SH2 and kinase domains is bound by the SH3 domain. Together, the SH2 and SH3 regulatory domains constrain the kinase in a state where the kinase domain is configured to restrict its catalytic activity. When the intramolecular interactions that stabilize the autoinhibited confirmation are disrupted, the kinase can adopt an active conformation that is stabilized by trans-autophosphorylation of its activation loop [10]. Although the domain and regulatory architecture of SFKs is conserved, SFKs can be divided into the SFK A and SFK B subfamilies by sequence conservation. SFK A is broadly expressed across tissues, whereas SFK B is generally restricted to the hematopoietic lineage. T cells express FYN and LCK, which belong to the SFK A and SFK B subfamilies, respectively, and interestingly, TIP was shown to bind preferentially to LCK versus FYN in T cells [1,11,12]. The shared homology between SFK B family members suggests that they could also bind TIP in immune cells.

TIP binds LCK using a proline-rich motif within TIP (SH3B) and an adjacent motif (CSKH) that shares homology with CSK [12]. Affinity measurements by fluorescence spectroscopy determined the binding affinity of a peptide derived from the proline-rich SH3B sequence to SH3 domains isolated from different SFKs. Notably, the SH3 domains of SFK B subfamily members LYN, HCK, and LCK possessed the highest affinities with dissociation constants (K_d_) between 2 and 10 μM [13]. The SH3 domains of SFK A subfamily members could also bind, but with comparatively higher dissociation constants. The LYN SH3 domain exhibited the lowest K_d_ and has been structurally resolved with a TIP-derived peptide [1]. A Minimal TIP variant that contained the SH3B and CSKH motifs was found to retain its ability to bind LCK in T cells [3]. It was determined that this Minimal TIP construct could serve as a platform for the recruitment of LCK to different STAT proteins in T cells to activate them in a cytokine-independent manner. Specifically, TIP possesses two STAT binding motifs (Y114 and Y127) that are phosphorylated by LCK, which together bind and broadly recruit multiple STAT proteins through their SH2 domains [2,14,15]. The recruited STATs are then phosphorylated by LCK on regulatory tyrosines that result in their activation. It was determined that the STAT binding motifs in the Minimal TIP variant could be replaced with those derived from cytokine receptors, such as the YLSLQ sequence derived from IL-2Rβ [16,17]. The Minimal IL-2Rβ variant (aSTAT5) was determined to activate STAT5 in primary T cells.

The association of aSTAT5 with LCK and STAT5, which exists as isoforms STAT5A and STAT5B, was determined by their co-immunoprecipitation from cell lysates [3,18,19]. However, it was undetermined whether the LCK or STAT5 binding motifs in aSTAT5 could interact with other binding partners in cells, or if other unknown interaction sites were present within aSTAT5. To evaluate these possibilities, we employed affinity purification mass spectrometry (AP-MS) to define the aSTAT5 interactome. Because AP-MS is an unbiased approach, we predicted that it would reveal any additional binding partners, as well as potential indirect interactions between aSTAT5 and T cell proteins. Because TIP can interact with LYN and potentially other SFK B subfamily members apart from LCK, we also investigated whether aSTAT5 could induce the phosphorylation of STAT5 in hematopoietic cells other than T cells. The SH3 domain is a conserved feature of SFKs found in lymphocyte populations like T and B cells, NK cells, and myeloid cells such as macrophages. We therefore expressed aSTAT5 in a panel of hematopoietic cell lines that express SFK B subfamily members that include LCK, HCK, LYN, and BLK. Interestingly, STAT5 activation was only observed in cells that expressed LCK, including the NK-92 natural killer (NK) cell line and primary human CD8+ T cells. Our findings suggest that LCK is necessary for aSTAT5-induced phosphorylation of STAT5 in cells and provide an assessment of its translational potential in human T cells.

## 2. Materials and Methods

### 2.1. Cell Lines

The AsPC-1 human cell line was obtained from ATCC (American Type Culture Collection, VA, USA). NK-92 cells were provided by the Tall lab (U-M, MI, USA). RAW 264.7 cells were obtained from the Lee lab (U-M, MI, USA). Jurkat T cells and Raji B cells were obtained from the Weiss lab (UCSF, CA, USA). Lenti-X 293T cell line was obtained from Takara Bio (CA, USA) and is denoted LX293T. Suspension cell lines were maintained in RPMI supplemented with 10% fetal bovine serum (FBS) and 2 mM glutamine. Adherent cell lines were maintained in DMEM culture medium with 10% FBS and 2 mM glutamine. NK-92 cells and primary human CD8+ T cells were cultured in complete RPMI [RPMI 1640 medium, 10% FBS, glutamine (2 mM), Penicillin/Streptomycin (Gibco, MA, USA), MEM Non-Essential Amino Acids (Gibco), 1 mM sodium pyruvate (Gibco), HEPES (10 mM, Corning (NY, USA), 2-Mercaptoethanol (50 μM, Gibco)] supplemented with recombinant human IL-2 (10 ng/mL). All cells were maintained in a tissue culture incubator at 37 °C with 5% CO_2_.

### 2.2. Antibodies and Reagents

Table S1 lists the antibodies used in this study with their respective concentrations. Reagents used in this study included the following: LR clonase (Thermo Fisher Scientific, MA, USA), QIAFilter plasmid purification kit (Qiagen, Venlo, The Netherlands), EasySep Human CD8 Positive Selection Kit II (StemCell Technologies, Vancouver, Canada), Anti-c-Myc Magnetic Beads (Thermo Fisher Scientific), TMTpro 16plex Label Reagent Set (Thermo Fisher Scientific), Halt protease inhibitor cocktail (Thermo Fisher Scientific), EDTA (Thermo Fisher Scientific), TransIT-LT1 transfection reagent (Mirus Bio, WI, USA), Opti-MEM (Gibco), RetroNectin (Takara Bio), NP-40 (EMD Millipore, MA, USA), recombinant human IL-2 (BioLegend), Ficoll-Paque PLUS (Cytiva, MA, USA), Zombie Violet fixable viability dye (BioLegend, CA, USA), propidium iodide (PI), Hoechst, and CFSE stains (Biolegend).

### 2.3. Plasmids

The aSTAT5 construct and its derivatives (Null and Minimal) in pEF6 backbones have been previously described [3]. For this study, a pHR EF1a gateway vector was generated by insertion of the gateway cassette flanked by an IRES and Thy1.1 into the multiple cloning sites. The gateway IRES Thy1.1 insert was PCR amplified and inserted into the BamHI and NotI restriction sites. A pDONR221 aSTAT5 plasmid and LR clonase (Thermo Fisher Scientific) were used to generate the final pHR EF1a aSTAT5 IRES Thy1.1 plasmid for lentiviral transduction. An empty vector with the same backbone as the respective aSTAT5 plasmid was used as a control. The lentiviral packing plasmid used was pCMV-dR8.91. Envelope plasmid (pMD2.G, no. 12259) and the mesothelin CAR plasmid (pOT_7-lenti-EFS-SS1-BBz-2A-puro, no. 217990) were obtained from Addgene (MA, USA).

### 2.4. Human CD8+ T Cell Purification

Peripheral blood samples from healthy human donors were obtained through the Platelet Pharmacology and Physiology Core at the University of Michigan Medical School. Whole blood was diluted 1:1 with phosphate-buffered saline (PBS) and underlaid with Ficoll-Paque PLUS. After centrifugation (400× *g*, 20 min, 20 °C, minimum acceleration and no brake), peripheral blood mononuclear cells (PBMCs) were separated out by a Ficoll density gradient. PBMCs were isolated and washed with FACS buffer [phosphate-buffered saline (PBS) with 2% FBS and 2 mM EDTA]. All solutions were warmed to room temperature (RT) prior to whole blood isolation. Primary human CD8+ T cells were purified from isolated PBMCs by positive selection using an EasySep Human CD8 Positive Selection Kit II (StemCell Technologies). Once purified, human CD8+ T cells were activated with plate-bound antibodies [anti-CD3 (5 μg/mL) and anti-CD28 (2.5 μg/mL)] in complete RPMI medium for 24 h in a tissue culture incubator at 37 °C before transduction.

### 2.5. Lentiviral Transduction

LX293T cells were cultured overnight in 6-well plates. The following day, LX293T cells were transfected with the packaging plasmids pCMV-dR8.2, pMD2.G, and a viral expression vector using TransIT-LT1 (Mirus Bio) according to the manufacturer’s instructions. After 18–24 h, the culture medium was replaced, and the viral supernatant was collected after 24 h and centrifuged to remove cells and debris before addition to 6-well plates at a volume of 1.5 mL per well. One mL of cell suspension (Jurkat, NK-92, or Raji) containing 10^5^ cells or fewer was then added and cultured with viral supernatants overnight. Adherent cells (RAW 264.7) were plated at 10^5^ 24 h prior to the addition of viral supernatant. Human CD8+ T cells were transduced using RetroNectin (15 μg/mL) adsorbed to non–TC-treated 6-well plates, which were then incubated with lentiviral supernatants at 37 °C for 1 h. Human CD8+ T cells were removed from plates following activation with anti-CD3/28 and washed, then combined with the viral supernatants and centrifuged (2000× *g*) at 37 °C for 1 h. Transduction efficiency was assessed across cell types 24 h post-transduction by flow cytometry to assess the proportion of Thy1.1+ cells. Total cells were counted using a Luna cell counter (Logos Biosystems, Anyang-si, South Korea), and viability was determined by staining with Zombie Violet followed by flow cytometry. Human CD8+ T cells were expanded in the presence of IL-2 for 2 days prior to cytokine washout by replacement with cytokine-free medium for 2–4 days prior to cell lysis or viability analysis.

### 2.6. Immunoblot Analysis

Cells were rinsed with their respective base media (without FBS) and resuspended at 20 × 10^6^ cells/mL before incubating at 37 °C for 15 min. Cells were lysed by the addition of ice-cold lysis buffer [1x dilution in TBS of 1% NP-40, EDTA (0.5 mM), NaF (10 mM), Na_3_VO_4_ (2 mM), and Halt protease inhibitor cocktail (100×)]. Lysates were placed on ice for 10 min before centrifugation (13,000× *g*) to remove debris at 4 °C. Lysates were combined with SDS sample buffer (6×) and incubated at 95 °C for 5 min before SDS-PAGE was performed using NuPAGE 4–12% Bis-tris gels (Invitrogen, CA, USA). Proteins were transferred to polyvinylidene difluoride (PVDF) membranes and incubated for 1 h at RT with TBS-T blocking buffer [tris-buffered saline (pH 7.4) and 0.1% Tween 20 supplemented with 3% bovine serum albumin (BSA)]. Blots were probed with primary antibodies indicated in Table S1, diluted in TBS-T blocking buffer and incubated overnight at 4 °C. Blots were washed 3x with TBS-T and incubated with horseradish peroxidase (HRP)-conjugated secondary antibodies (1:5000 in TBS-T blocking buffer) for 1 h at RT. Blots were imaged using Pierce ECL chemiluminescent substrate (Thermo Fisher Scientific) and an iBright (Invitrogen) system, followed by quantification with iBright analysis software version 5.2.0 (Invitrogen).

### 2.7. NK/T Cell-Mediated Cytotoxicity

NK cell and CD8+ human T cell-mediated cytotoxicity were assessed as previously described [20]. Raji and AsPC-1 cancer cells were stained with 1–2.5 µM CSFE for 20 min at 37 °C and added to round-bottom 96-well plates in phenol red-free complete MEM at a density of 25,000 cells per well. NK-92 and T cells were transduced as outlined above and treated with cytokine or subjected to a washout (24–48 h) before being added at different effector-to-target (E:T) ratios and co-cultured for 18, 24, or 48 h at 37 °C. For human CD8+ T cells experiments, AsPC-1 cells were plated 24 h prior to co-culture at a density of 5000 cells per well in a flat-bottom plate. Immediately prior to imaging, all cells were stained with PI (0.5 mg/μL) and Hoechst dye (20 μM) for 30 min at 37 °C. Plates were then imaged using a Celigo imaging cytometer (PerkinElmer, MA, USA) to quantify dead cancer cells (CFSE+, PI+). Cancer cell-only controls were used to calculate effector cell-mediated cytotoxicity. Data were analyzed using Celigo analysis software version 5.3.

### 2.8. Flow Cytometry

Prior to flow cytometry analysis, cells were removed from culture and washed twice with FACS buffer. They were then stained with the Zombie Violet (Biolegend) fixable live/dead stain (1:1000 in PBS) at RT for 15 min. Cells were then washed with FACS buffer and incubated with any additional antibodies (Table S1). Antibodies were diluted in FACS buffer at 1:200 for 30 min on ice. Cells were then washed twice with ice-cold FACS buffer prior to analysis on a CytoFLEX flow cytometer (Beckman Coulter, CA, USA). Transduction was assessed at 24 h following. Cell viability was analyzed after 3, 4, 6, and/or 9 days in culture as indicated in figure captions. Following collection, the data were analyzed using FlowJo software version 10.10.0 (BD Biosciences, CA, USA). Gating strategies are provided in Figure S3.

### 2.9. Mass Spectrometry

Jurkat T cells were washed and resuspended in RPMI. The pEF6 aSTAT5 plasmid (10 μg) was combined with 15 × 10^6^ cells in 400 μL in a 0.4 cm cuvette and then electroporated using a Bio-Rad Gene Pulser Xcell (CA, USA) (260 V, 1250 μF) at RT. Cells were recovered into RPMI culture medium and incubated at 37 °C for 48 h. Cells were rinsed with RPMI and resuspended at 15 × 10^6^ cells/mL before incubating at 37 °C for 15 min. Cells were then lysed as above at 15 × 10^6^ cells/mL, and total protein concentrations were determined using a BCA assay. For immunoprecipitation, Jurkat T cell lysates were combined with 50 μL of anti-MYC tag (9E10) beads (Pierce, CA, USA). Samples were mixed by rotation for 2 h at 4 °C before washing four times with ice-cold wash buffer (1/10 lysis buffer diluted with TBS), and proteins were eluted by adding SDS sample buffer. Aliquots of the samples were set aside for QC via Western blot. Following confirmation of successful immunoprecipitation as indicated by the detection of the Myc tag via Western blot, samples were submitted to the Proteomics Resource Facility at the University of Michigan for processing and mass spectrometry data acquisition. Proteins were precipitated from beads using 6 volumes of ice-cold acetone followed by overnight incubation at −20 °C. Samples then underwent overnight protein digestion at 37 °C with 1 mg trypsin/Lys-C mix (Promega, WI, USA) and were resuspended in 100 μL of 100 mM TEAB, pH 8.5. Samples were then labeled with the TMTpro reagents (Thermo Fisher Scientific) and pooled. Three replicates of beads (EV), Null (SH3B CSKH), Minimal (TIP MIN), and aSTAT5 (STAT5a) were labeled with TMT channels 126, 127N, 128N, 129N, 130N, 131N, 132N, 133N, 134N, 127C, 128C, 129C respectively. An offline fractionation of the combined sample (~200 g) into 8 fractions was performed using a high pH reversed-phase peptide fractionation kit according to the manufacturer’s protocol (Thermo Fisher Scientific, 84868). The combined sample was reconstituted in 600 µL 0.1% formic acid/2% acetonitrile in preparation for LC-MS/MS analysis. Labeled samples were analyzed on an Orbitrap Ascend Tribrid equipped with a FAIMS source (Thermo Fisher Scientific, MA, USA) and Vanquish Neo UHPLC. Two μL of the sample was resolved on an Easy-Spray PepMap Neo column (75 μm i.d. × 50 cm; Thermo Scientific, MA, USA) at a flow rate of 300 nL/min using 0.1% formic acid/acetonitrile gradient system (3–19% acetonitrile in 72 min; 19–29% acetonitrile in 28 min; 29–41% in 20 min followed by 10 min column wash at 95% acetonitrile and re-equilibration) and directly sprayed onto the mass spectrometer using an EasySpray source (Thermo Fisher Scientific). FAIMS source was operated in standard resolution mode, with a nitrogen gas flow of 4.2 L/min, and inner and outer electrode temperatures of 100 °C and dispersion voltage of −5000 V. Two compensation voltages (CVs) of −45 and −65 V, 1.5 s per CV, were employed to select ions that enter the mass spectrometer for MS1 scan and MS/MS cycles. Mass spectrometer was set to collect MS1 scan (Orbitrap; 400–1600 *m*/*z*; 120 K resolution; AGC target of 100%; max IT in Auto) following which precursor ions with charge states of 2–6 were isolated by quadrupole mass filter at 0.7 *m*/*z* width and fragmented by collision induced dissociation in ion trap (NCE 30%; normalized AGC target of 100%; max IT 35 ms). Real Time Search (RTS) option with the human database was used to select peptides for MS3 analysis using Proteome Discoverer software, version 3.0 on the MS2 spectra (Thermo Fisher Scientific, V3.0) using the following parameters: MS1 and MS2 tolerance were set to 10 ppm and 0.6 Da, respectively; carbamidomethylation of cysteines (57.02146 Da) and TMT labeling of lysine and N-termini of peptides (229.16293 Da) were considered static modifications; oxidation of methionine (15.9949 Da) and deamidation of asparagine and glutamine (0.98401 Da) were considered variable. Identified proteins and peptides were filtered to retain only those that passed a ≤1% FDR threshold. Quantitation was performed using high-quality MS3 spectra (Average signal-to-noise ratio of 10 and <50% isolation interference). Further statistical tests of the quantification were done with the normalized raw abundances in the Perseus software platform, version 2.1.6.0 [21].

### 2.10. Statistical Analysis

All statistical analyses outside AP-MS data were performed using GraphPad Prism software (V10.4.1), and data are shown as mean with error bars denoting the standard deviation (SD). Significance was determined by analysis of variance (ANOVA), and *p* values are indicated by the following: * *p* < 0.05, ** *p* < 0.01, *** *p* < 0.001, and **** *p* < 0.0001; *p* > 0.05 was considered not significant (ns). The number of independent experiments is denoted as n within the figure captions.

## 3. Results

### 3.1. Characterization of the aSTAT5 Interactome by Affinity Purification Mass Spectrometry (AP-MS)

**Figure 1:**
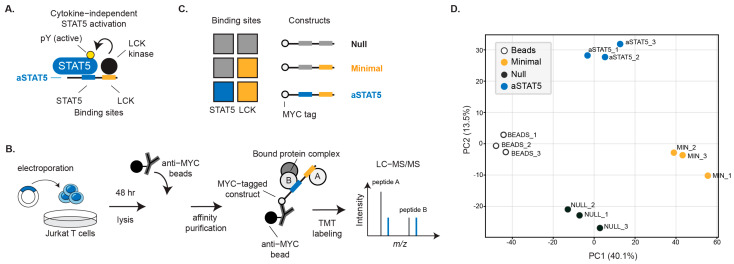
Characterization of the aSTAT5 interactome by affinity purification mass spectrometry (AP-MS). (**A**) aSTAT5 binds LCK to phosphorylate and activate endogenous STAT5. (**B**) Jurkat T cells were transiently transfected to express TIP variants for analysis by AP-MS. After 48 h, cells were lysed, and anti-MYC beads were used to immunoprecipitate proteins associated with the MYC-tagged constructs. Proteins were identified by quantitative mass spectrometry (MS). (**C**) Binding sites present within the aSTAT5, Null, and Minimal constructs. (**D**) PCA of normalized abundance values for individual samples obtained by LC-MS/MS (*n* = 3).

aSTAT5 was engineered from a Minimal HVS TIP variant and contains LCK binding motifs and a STAT5 binding motif derived from the IL-2 receptor β-chain (IL-2Rβ) (Figure 1A). Because these motifs could interact with other SH2 and SH3 domain-containing proteins, such as related kinases or STATs, we sought to define aSTAT5 interaction partners in an unbiased manner. The aSTAT5 interactome was therefore assessed by AP-MS to determine proteins that bind aSTAT5 or are indirectly associated with it (Figure 1B). In order to assess which aSTAT5 interactions are mediated by its specific binding motifs, a comparative analysis was performed using mutational variants of aSTAT5. Specifically, mutation of an essential tyrosine residue to alanine disrupted a STAT binding motif to yield a Minimal construct that retained only its LCK binding motifs (CSKH and SH3B). A Null variant was also generated where both the STAT5 and LCK binding motifs were disrupted in combination. LCK binding to the Null variant was prevented by mutation of key residues within the SH3 and CSKH motifs to alanine (Figure 1C) [22]. Each TIP variant contained an N-terminal MYC tag to facilitate its affinity purification from cell lysates and capture associated proteins. Jurkat T cells were transiently transfected to express aSTAT5 and its variants, then cultured for 48 h prior to lysis and affinity purification using anti-MYC beads. Samples, as well as a beads-only control, were labeled with tandem mass tags (TMT), and the proteins associated with each construct were identified by quantitative mass spectrometry (MS).

**Figure 2:**
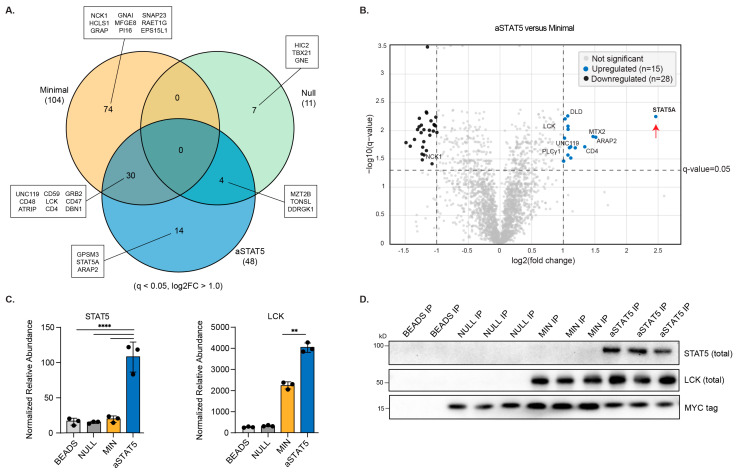
Analysis of the aSTAT5 interactome by AP-MS. (**A**) Venn diagram of identified proteins (q < 0.05, log2FC > 1.0) obtained from the Minimal, Null and aSTAT5 samples. Proteins with the highest log2FC for each comparison are boxed and annotated, depicted in descending rank order. (**B**) Volcano plot of differentially enriched proteins between aSTAT5 and Minimal (increased enrichment q < 0.05, log2FC > 1.0 are denoted in blue; reduced enrichment (q < 0.05, log2FC < −1.0 are denoted in black). (**C**) Normalized relative abundance of STAT5 and LCK in sample groups (*n* = 3, means ± SD); ** *p* < 0.01; **** *p* < 0.0001; two-way ANOVA, Šidák’s multiple comparisons. (**D**) Immunoblot analysis of proteins co-immunoprecipitated from Jurkat T cell lysates (*n* = 3).

Principal component analysis (PCA) of the datasets resulted in distinct clustering of samples that corresponded to each construct. Independent replicates of each construct clustered tightly together, indicating a limited batch effect. Clusters were comparatively closer between the Minimal and Null constructs, indicating more similar sample composition, whereas the aSTAT5 was more separated (Figure 1D). Normalized abundance values for individual samples underwent permutation-based FDR control to calculate protein enrichment. This dataset was used to compare proteins associated with aSTAT5 versus the Minimal variant, which differs only by its disrupted STAT5 binding motif, by determining their fold-change. Proteins that were enriched by aSTAT5 versus Minimal included membrane associated proteins like ARAP2, UNC119, and CD4 (Figure 2A, Table S2). Notably, STAT5A displayed the greatest enrichment (>5 fold-change) in comparison to Minimal (Figure 2B, Table S2), consistent with its interaction with the IL-2Rβ-derived STAT5 binding motif. Similarly, STAT5 was noticeably absent when compared to Null and the beads-only control (Figure 2C). LCK enrichment was observed when Minimal and aSTAT5 were compared to the Null construct, which was expected given the LCK binding motifs within Minimal and aSTAT5, which were not in the Null construct (Figure 2C) [1,23]. Interestingly, LCK was enriched by aSTAT5 to a greater extent than Minimal despite both constructs possessing an identical LCK binding motif (Figure 1C). LCK and STAT5 association was validated across the panel of constructs by co-immunoprecipitation (Figure 2D). T cell receptor signaling (TCR)-associated proteins were enriched by both the Minimal and aSTAT5 constructs, which included PLCγ, GRB2, and GRAP, as well as CSK, a negative regulator of LCK (Figure 2A, Table S3). Because aSTAT5 is derived from TIP, which is known to bind other STATs such as STAT3 and STAT6, the extent to which these other STATs were enriched by aSTAT5 was evaluated (Table S3); only STAT5 was significantly enriched within the dataset. As the Null contains mutationally disrupted binding motifs, we see fewer proteins associated when compared to Minimal and aSTAT5, as expected (Figure 2A). When the Null construct was compared to the beads-only control, negligible protein enrichment (>Log2FC1 cutoff) was observed (Figure S1A). Overall, these observations are consistent with the LCK binding motifs and the IL-2Rβ-derived STAT5 binding motif being required to mediate an interaction with STAT5A.

### 3.2. STAT5 Activation in Hematopoietic Cell Lines

**Figure 3:**
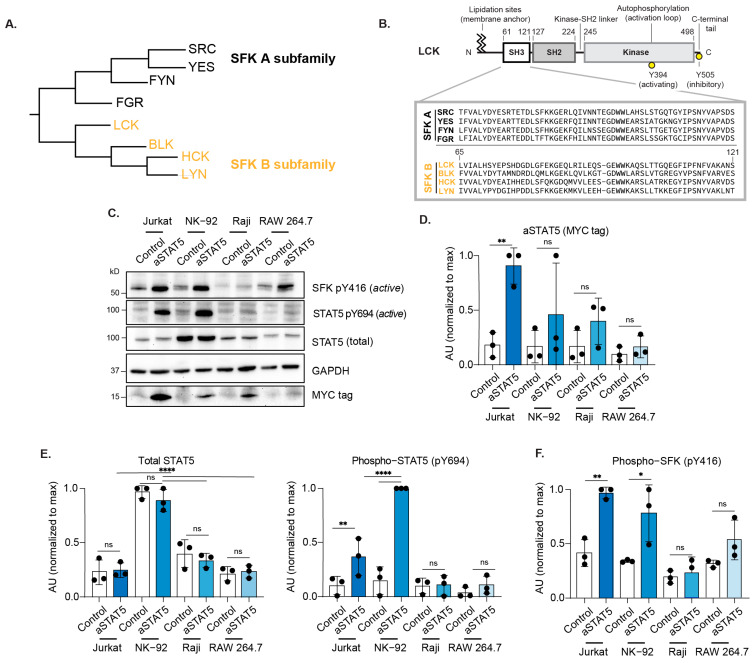
STAT5 activation in hematopoietic cell lines. (**A**) Dendrogram depicting the SRC family kinase (SFK) A and B subfamilies. (**B**) Schematic depicting the domain architecture of the SFK LCK and sequence alignment of SFK B SH3 domains. (**C**) Immunoblot analysis of phosphorylated STAT5 and SFKs (activation loop: SRC Y416, LCK Y394). Control denotes an empty vector. Immunoblot analysis is representative of three independent experiments (*n* = 3). (**D**) Quantification of aSTAT5 levels (MYC tag), (**E**) Phospho-STAT5 and total STAT5, and (**F**) Phospho-SFK (activation loop: SRC Y416, LCK Y394) (means ± SD); ns = *p* > 0.05; * *p* < 0.05; ** *p* < 0.01; **** *p* < 0.0001; two-way ANOVA, Šidák’s multiple comparisons (*n* = 3).

The SFK B subfamily (LYN, HCK, BLK, and LCK) possesses a highly conserved SH3 domain (Figure 3A,B). Because the LCK SH3 domain mediates binding to the SH3B region of TIP and is required for LCK-induced phosphorylation of recruited STAT proteins, we sought to assess whether aSTAT5 expression could induce STAT5 phosphorylation in human hematopoietic cell lines that express LCK and related SFKs (Figure 3B and Figure S1B, Table S4). The NK-92 natural killer (NK) cell line, RAW 264.7 macrophage cell line, Raji B cell line, and Jurkat T cell line were transduced using a lentiviral vector to express either aSTAT5 or control (empty vector). Transduction efficiency was assessed 24 h post-transduction by flow cytometry using the surface marker Thy1.1 and determined to be >80% across each of the cell lines (Figure S1C). Whole cell lysates were prepared, and the extent of STAT5 phosphorylation was determined by immunoblot. Because NK-92 cells require the cytokine IL-2 to maintain their viability, IL-2 was removed from NK-92 cell cultures 48 h prior to lysis. To assess differences in aSTAT5 expression across cell lines, aSTAT5 levels were assessed by blotting for its MYC tag (Figure 3C,D). aSTAT5 levels were greatest in Jurkat cells, with the lowest levels observed in RAW 264.7 cells. We observed aSTAT5 expression induced robust phosphorylation of STAT5 in Jurkat T cells, which was consistent with prior results obtained by transient transfection [3]. Notably, STAT5 was phosphorylated to the greatest extent in NK-92 cells that expressed aSTAT5 (Figure 3C,E). Unexpectedly, given the similarity between LCK and LYN or HCK, STAT5 phosphorylation was not appreciably induced in Raji cells by aSTAT5 or in the RAW 264.7 cell line (Figure 3C,F). We observed increased phosphorylation of the conserved activating SRC Y416 regulatory site of SFKs expressed by Jurkat, NK-92 and RAW 264.7 cells. Whereas this increased SFK activity corresponded to increased STAT5 phosphorylation in Jurkat and NK-92 cells, a similar increase was not observed in RAW 264.7 cells. These findings demonstrate that, similar to T cells, aSTAT5 can induce the phosphorylation of STAT5 in NK-92 cells.

### 3.3. NK-92 Cell Viability and Proliferation Is Sustained by aSTAT5

**Figure 4:**
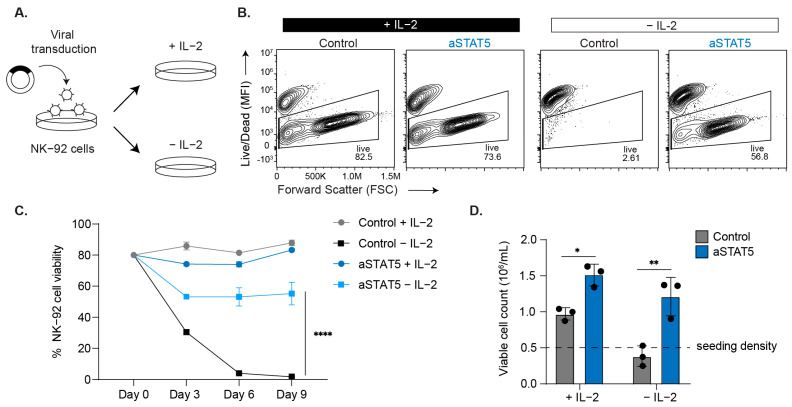
aSTAT5 maintains natural killer cells’ viability and proliferation in the absence of IL-2. (**A**) NK-92 cells were transduced to express aSTAT5 or control (empty vector) and then cultured in the presence or absence of IL-2 (10 ng/mL). (**B**) Representative data depicting NK-92 cell viability assessed by flow cytometry on day 6 post-transduction. (**C**) Quantification of NK-92 cell viability on 3, 6, and 9 days post-transduction (*n* = 3, means ± SD); **** *p* < 0.0001; two-way ANOVA, Šidák’s multiple comparisons. (**D**) Quantification of NK-92 cell proliferation 6 days post-transduction (means ± SD); * *p* < 0.05; ** *p* < 0.01; two-way ANOVA, Šidák’s multiple comparisons (*n* = 3).

NK cells require JAK-STAT signaling induced by the cytokines IL-15 and IL-7 for homeostasis and IL-12 and IL-2 to undergo expansion [24]. It was previously observed that the conditional deletion of STAT5 in NK cells resulted in the complete loss of peripheral NK cells in mice [25,26]. We therefore evaluated whether aSTAT5 could replace the cytokine-induced STAT5 signaling required for NK cell survival using the NK-92 cell line, which depends on IL-2 in culture for their survival and expansion. To assess whether aSTAT5 could maintain NK-92 cell viability, cells were transduced to express either aSTAT5 or a control (empty vector) and then cultured in the presence or absence of IL-2 (Figure 4A). Viability was assessed after 3, 6, and 9 days in culture by flow cytometry. NK-92 cells that were cultured with IL-2 cytokine were >78% viable (Figure 4B and Figure S3A), whereas a marked drop in viability occurred by day 3 when control cells were cultured without IL-2. By contrast, NK-92 cells that expressed aSTAT5 remained viable in the absence of IL-2 to a similar extent to cells cultured with IL-2 (Figure 4B). aSTAT5 maintained NK-92 cell viability over 9 days, where approximately 60% of cells remained viable (Figure 4C). NK-92 cells cultured with IL-2 underwent a robust expansion by day 6, which was similarly observed when cells expressed aSTAT5 even in the absence of IL-2 (Figure 4D). Interestingly, aSTAT5 expression resulted in greater NK-92 expansion than control, both in the presence or absence of IL-2.

### 3.4. Cytokine-Independent STAT5 Activation in Human CD8+ T Cells Is Sufficient to Maintain Their Viability Ex Vivo

**Figure 5:**
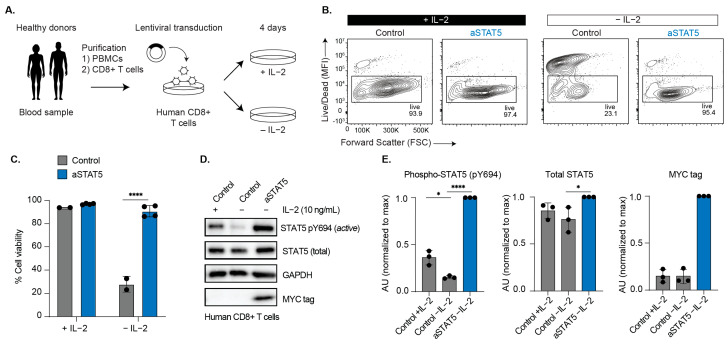
aSTAT5 maintains primary human CD8+ T cell viability in the absence of IL-2. (**A**) PBMCs were obtained from the blood of healthy human donors, followed by isolation of primary human CD8+ T cells. Human CD8+ T cells were activated using anti-CD3 and anti-CD28 antibodies and transduced with aSTAT5 or control (empty vector), then cultured in the presence or absence of IL-2 for 4 days. (**B**) Representative data depicting T cell viability assessed by flow cytometry on day 4 post-transduction. (**C**) Quantification of human CD8+ T cell viability following 4 days of +/− cytokine treatment (means ± SD); **** *p* < 0.0001; two-way ANOVA, Šidák’s multiple comparisons (*n* = 2–4). (**D**) aSTAT5 was assessed for its capacity to induce STAT5 phosphorylation in human CD8+ T cells by immunoblot analysis. IL-2 was removed 48 h prior to lysis of control (−IL-2) (*n* = 3). (**E**) Quantification of pSTAT5, total STAT5, and aSTAT5 (MYC tag) levels from immunoblot analysis (*n* = 3, means ± SD); * *p* < 0.05; **** *p* < 0.0001; two-way ANOVA, Šidák’s multiple comparisons.

Mouse models were previously used to demonstrate that cytokine-independent activation of aSTAT5 could maintain CD8+ T cell function [3]. We therefore used primary human CD8+ T cells to evaluate the translational potential of aSTAT5 by determining whether it could maintain their viability in the absence of IL-2 ex vivo. PBMCs were isolated from the blood of healthy human donors using a Ficoll gradient followed by bead-based positive selection to isolate CD8+ T cells. CD8+ T cells were then activated using plate-bound anti-CD3 and anti-CD28 antibodies and transduced with aSTAT5 or control (empty vector) (Figure 5A). The transduced cells were cultured in the presence or absence of IL-2, and their viability was determined by flow cytometry. Human CD8+ T cells remained over 90% viable when cultured in medium supplemented with IL-2; however, when IL-2 was removed, we observed markedly decreased viability by day 4 (Figure 5B,C and Figure S3B). When cells expressed aSTAT5, their viability was markedly increased in the absence of IL-2 (Figure 5B,C). To assess the relationship between viability and STAT5 activation, we evaluated the extent of STAT5 phosphorylation by immunoblot (Figure 5D). We observed that aSTAT5 induced robust phosphorylation of STAT5 to a greater extent than IL-2 treatment (Figure 5D,E). These data were consistent with previous observations in murine models and suggest that aSTAT5 could also be used to maintain human CD8+ T cell function in settings where cytokines like IL-2 are depleted.

### 3.5. NK-92 and Human CAR T Cell Functionality Is Sustained in Culture by aSTAT5

**Figure 6:**
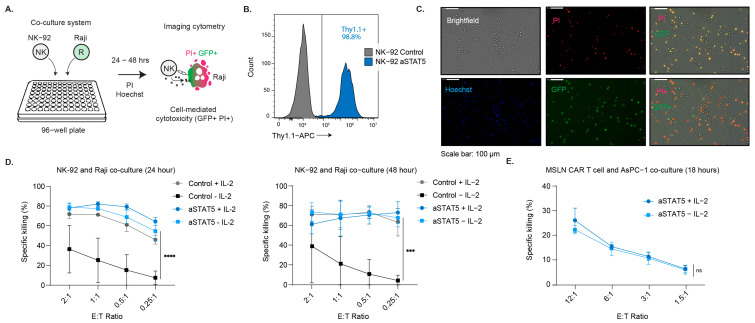
NK-92 and CAR-T cell cytotoxicity is maintained in the absence of IL-2. (**A**) The capacity of aSTAT5 to sustain NK-92 cell-mediated cytotoxicity in the absence of IL-2 was assessed by co-culturing NK-92 cells with CFSE-stained Raji cancer cells for 24 or 48 h. (**B**) aSTAT5 or control (empty vector) was stably transduced into NK-92 cells by lentiviral transduction. The percentage of the population expressing aSTAT5 as indicated by Thy1.1, was assessed by flow cytometry. (**C**) Representative data depicting the extent of cancer cell death (PI+, GFP+) as determined by imaging cytometry. Scale bar is 100 µm. (**D**) Quantification of NK-92 cell-mediated cytotoxicity at different E:T ratios taken at 24 and 48 h. Cytokine was included or washed out for 48 h prior to co-culture, where indicated. (*n* = 3, means ± SD); *** *p* < 0.001; **** *p* < 0.0001; two-way ANOVA, Šidák’s multiple comparisons. (**E**) Quantification of human MSLN CAR T cell-mediated cytotoxicity in the absence of cytokines at different E:T ratios after 18 h of co-culture. Cytokine was included or washed out for 24 h prior to co-culture, where indicated. (*n* = 2, means ± SD); ns = *p* > 0.05; two-way ANOVA, Šidák’s multiple comparisons.

NK-92 can recognize and eliminate target cells using a variety of receptors, including NKG2D and DNAM-1 [27,28,29]. NKG2D detects ULBP and UL16 binding proteins, whereas DNAM-1 detects CD155, CD112, or Nectin-2. Raji B cells have been shown to express these ligands and are therefore recognized and killed by NK-92 cells in culture [30]. To assess NK-92 killing of Raji B cells ex vivo in the absence of IL-2, we utilized a co-culture assay followed by imaging cytometry to observe specific NK-92 cell-mediated cytotoxicity (Figure 6A). IL-2-containing medium was removed from NK-92 cells that expressed aSTAT5 and control (empty vector) 24–48 h prior to co-culture with Raji B cells at various E:T ratios (Figure 6B and Figure S3C). Raji B cells were labeled with CFSE to identify the target cell population. Following 24–48 h of co-culture, cells were stained with PI and Hoechst and analyzed by imaging cytometry (Figure 6C). After 24 h of co-culture, we observed robust cell-specific killing across E:T ratios (Figure 6D). Notably, aSTAT5 could maintain the functional capacity of NK-92 cells to kill Raji cancer cells to a similar degree as cells cultured with IL-2. IL-2 and aSTAT5 resulted in NK-92-mediated cytotoxicity at E:T of 2:1 and 1:1, with greater than 70% and above 50% at 0.25:1. When assessed at 48 h, the same co-cultures displayed >60% killing for all E:T ratios when cells were transduced with aSTAT5 cultured with IL-2 (Figure 6D). By contrast, when control cells lacked IL-2, their functional capacity to kill Raji cells was markedly reduced. Because aSTAT5 promoted human CD8+ T cell viability, we assessed whether it could enhance CAR T cell cytotoxicity in the absence of IL-2. Human CD8+ T cells were transduced to express a mesothelin (MSLN)-specific CAR and aSTAT5 (Figure S2A,B). CAR T cells were expanded, and IL-2 was removed (−IL-2) 24 h prior to co-culturing with mesothelin-expressing AsPC-1 pancreatic ductal adenocarcinoma (PDA) cells. After 18 h, CAR-T cell-mediated cytotoxicity was determined by assessing AsPC-1 viability (Figure 6E). CAR T cells that expressed aSTAT retained their capacity to eliminate the AsPC-1 cell line, comparable to those cultured with IL-2.

## 4. Discussion

The herpesvirus saimiri tyrosine kinase interacting protein was previously engineered to develop a cytokine-independent STAT5 activator (aSTAT5) [3]. aSTAT5 was demonstrated to recruit STAT5 through a sequence derived from the IL-2Rβ chain and subsequently induce its phosphorylation in an LCK kinase-dependent manner [2,3,11,17,22,31]. Here, we employed proteomics to define the aSTAT5 interactome by AP-MS, which confirmed that the STAT5 binding motif derived from IL-2Rβ acts to predominantly recruit STAT5 and not other related STAT proteins. Interestingly, when the extent of LCK association was compared between the aSTAT5 and Minimal construct by AP-MS, which contained an identical LCK binding region and only differed by its lack of a STAT5 binding motif, the amount of LCK association was modestly but reproducibly increased, suggesting that the association between the TIP variant and LCK was stabilized further by association with STAT5 or the STAT5 binding motif itself. The aSTAT5 interactome was also determined by AP-MS to contain TCR signaling proteins that included PLCγ, GRB2, and GRAP. When compared to the Minimal and Null constructs, many interactions were determined to be LCK dependent. Consistent with the indirect recruitment of proteins by LCK, CD4, which is known to bind to LCK through its N-terminal region, was associated with aSTAT5 and Minimal, but its interaction was lost from the Null construct, where the LCK binding motif was disrupted. Similarly associated with both aSTAT5 and Minimal but not Null was UNC119, which possesses two proline-rich motifs and is an SH3 domain ligand that has been previously observed to co-immunoprecipitate with CD4 and LCK [32]. GRB2 and GRAP are adapter proteins that contain SH2 and SH3 domains and motifs that mediate protein–protein interactions [33,34]. GRB2 and GRAP were found to be more strongly associated with Minimal than the aSTAT5 construct and absent from Null. Interestingly, the SH3 domain of PLCγ was not previously observed to bind to the WT TIP, and its binding is absent from Null, which suggests that its interaction is likely to be LCK dependent [13]. Overall, the interactome differed between these closely related constructs based on the presence of binding motifs that mediated direct interactions with LCK and STAT5 as well as indirect interactions, particularly with the kinase LCK.

When aSTAT5 was evaluated using a panel of human hematopoietic cell lines for its ability to disrupt SFK autoinhibition, increased SFK phosphorylation within the conserved activation loop (LCK Y394, SRC Y416) was observed in Jurkat and NK-92 cells that expressed LCK, and also the RAW 264.7 cell line that did not. These observations suggest that in a cellular context, the LCK binding motifs present within TIP favor LCK but can also interact with other closely related SFKs. Interestingly, the Raji B cell line did not appear to undergo an appreciable increase in SFK activity that would be characteristic of aSTAT5 binding. This observation was unexpected given the similarity between LYN and LCK, as well as previous studies demonstrating the ability of LYN to bind a peptide derived from the SH3B region of TIP [35]. These observations suggest some closely related SFKs may be more likely to interact with aSTAT5 than others, perhaps due to the contribution of the less well-defined CSKH motif, or their effects on SFK activity could be partly dependent on the cellular context, such as the levels of SFKs and the activities of their regulators like CSK and CD45 [36]. Despite the increased SFK activity observed in the RAW 264.7 cell line, only the LCK-expressing Jurkat and NK-92 cells displayed increased STAT5 phosphorylation. It is unclear why STAT5 phosphorylation was not induced by aSTAT5 in RAW 264.7 cells, but it could be attributed to lower levels of both aSTAT5 and STAT5. The level of STAT5 appears to be an important determinant, as the amount of phosphorylated STAT5 was greatest in NK-92 cells, which possessed the highest levels of STAT5. Indeed, this robust STAT5 phosphorylation occurred despite lower levels of aSTAT5 in comparison to the Jurkat T cell line. A potential implication of this finding is that more abundant STAT proteins in RAW 264.7 cells might be more readily activated by TIP variants with alternative STAT binding motifs. Whereas the Raji B cell line has similar levels of STAT5 to Jurkat, the inability of aSTAT5 to induce STAT5 phosphorylation in this cell line is likely due to a lack of SFK binding or activity. The induction of STAT5 phosphorylation in the human Jurkat T cell line extended to primary human CD8+ T cells, suggesting that the mechanism of STAT5 activation is likely dependent on the characteristic kinases and STAT5 levels of each cell line and would therefore be predicted to translate to primary cells that possessed similar SFK profiles and STAT levels.

The induction of STAT5 phosphorylation by aSTAT5 resulted in the survival of NK-92 and human CD8+ T cells in the absence of IL-2 ex vivo. Donor-derived human NK cells have been evaluated in clinical trials as an adoptive cellular immunotherapy for cancer treatment [37]. Primary human NK cells recognize targets through the expression of Fc, NKp30, NKp44, NKG2D, DNAM-1, and MHC I receptors, the latter to identify and kill “non-self” targets [27,28,29], and can be engineered for the development of CAR NK cell therapies [38]. NK-92 cells have also been developed as an off-the-shelf product for the treatment of cancer to address challenges with collecting sufficiently high numbers of primary human NK cells [37]. NK-92 cells require irradiation prior to transfer, which is deleterious to their survival [30], and have been co-administered with pro-survival cytokines like IL-2 and IL-15 to boost their persistence in vivo [39,40,41]. CARs directed against mesothelin (MSLN), a tumor antigen commonly upregulated in pancreatic ductal adenocarcinoma (PDA), have shown promise, but their efficacy in human clinical trials has been limited [42,43]. A major barrier to the success of NK and T cell therapies has been the lack of persistence and functionality of tumor-targeted cells in solid tumors, due to a highly suppressive tumor microenvironment (TME) that lacks pro-survival cytokines [44]. NK and T cell-based therapies have been administered with cytokine receptor agonists to promote their survival within the hostile TME [39,45,46]. Although the persistence or expansion of transferred cells was increased by cytokine administration, these extracellular signals can still be rapidly cleared or provide insufficient efficacy [39,46,47]. The cytokine-independent activation of STAT5 provides an alternative strategy that could be combined with NK and T cell therapies. Notably, mouse models demonstrated that cytokine-independent activation of STAT5 prevented tumor-specific T cells from acquiring hallmarks of T cell exhaustion in the B16-OVA model [3]. These data are consistent with observations using a constitutively active STAT5 mutant (STAT5CA) that was found to improve the ability of tumor-specific T cells to control tumor growth in mouse models [48,49,50]. Activation of STAT5 has also been accomplished using synthetic cytokine receptors that recognize orthogonal cytokines and are constitutively active [51,52]. The signals provided by these synthetic constructs were used to tailor effector CAR T cells with potent anti-tumor activity through the optimization of T cell phenotypes [53].

Here, we demonstrate that aSTAT5 can induce the phosphorylation of STAT5 in primary human CD8+ T cells, which maintained their viability ex vivo, as well as the cytotoxic capacity of MSLN-targeted CAR T cells. These observations suggest that aSTAT5 could equip CAR T cells with a STAT5 signal to enhance their persistence and function in solid tumors; however, the ex vivo co-culture systems evaluated do not recapitulate the full complexity of the TME. This limitation of our study provides a rationale for analysis of aSTAT5 using primary human NK cells and CAR T cells combined with relevant disease models of autoimmunity and refractory solid tumors. These models would provide a more comprehensive analysis of a cytotoxic response, including IFN-γ and TNF-α production, as well as alterations to immune cell phenotypes. As a therapeutic strategy, an additional consideration is the potential for STAT5 activity to promote the lymphoproliferative disease, and thus, aSTAT5 should be evaluated with integrated control mechanisms to mitigate this risk, such as kill switches [54,55]. Overall, here we characterize a strategy for the cytokine-independent activation of STAT5 that can sustain the viability and function of NK-92 cells and primary human CD8+ T cells.

## Data Availability

Data are provided in the article and Supplemental Files.

## Supplementary Materials

The following supporting information can be downloaded at: https://www.mdpi.com/article/10.3390/biomedicines14051097/s1, Figure S1. Additional comparisons of AP-MS controls and transduction efficiency; Figure S2. Human CD8+ T cells transduced to express a mesothelin (MSLN)-specific CAR; Figure S3. Quantification of cell viability and transduction efficacy; Table S1. Antibodies used; Table S2. Top enriched proteins; Table S3. Additional proteins of interest; Table S4. SRC family kinases expressed in immune cells.

## Abbreviations

The following abbreviations are used in this manuscript:

HSV	Herpesvirus saimiri
TIP	Tyrosine kinase interacting protein
SFK	SRC family kinase
aSTAT5	Cytokine-independent STAT5 activator
AP-MS	Affinity purification mass spectrometry
NK	Natural killer
MSLN	Mesothelin
CAR	Chimeric antigen receptor
IL-2Rb	IL-2 receptor β-chain
TCR	T cell receptor
BCR	B cell receptor
FcR	Fc receptor
ITAM	Immunoreceptor tyrosine-based activation motif
SH3B	Proline-rich SH3 binding motif
CSKH	CSK homology motif
TMT	Tandem mass tags
MS	Mass spectrometry
PCA	Principal component analysis
PBMC	Peripheral blood mononuclear cells
PI	Propidium iodide
CFSE	Carboxyfluorescein succinimidyl ester
PDA	Pancreatic ductal adenocarcinoma
TME	Tumor microenvironment

## References

[1] Bauer F., Hofinger E., Hoffmann S., Rösch P., Schweimer K., Sticht H. (2004). Characterization of Lck-Binding Elements in the Herpesviral Regulatory Tip Protein. Biochemistry.

[2] Lund T.C., Garcia R., Medveczky M.M., Jove R., Medveczky P.G. (1997). Activation of STAT transcription factors by herpesvirus Saimiri Tip-484 requires p56lck. J. Virol..

[3] Zheng Y., Gu Z., Shudde C.E., Piper T.L., Wang X., Aleck G.A., Zhou J., King D., Chanda M.K., Trinch L. (2025). An engineered viral protein activates STAT5 to prevent T cell suppression. Sci. Immunol..

[4] Kingston D., Chang H., Ensser A., Lee H.-R., Lee J., Lee S.-H., Jung J.U., Cho N.-H. (2011). Inhibition of Retromer Activity by Herpesvirus Saimiri Tip Leads to CD4 Downregulation and Efficient T Cell Transformation. J. Virol..

[5] Min C.-K., Bang S.-Y., Cho B.-A., Choi Y.-H., Yang J.-S., Lee S.-H., Seong S.-Y., Kim K.W., Kim S., Jung J.U. (2008). Role of Amphipathic Helix of a Herpesviral Protein in Membrane Deformation and T Cell Receptor Downregulation. PLoS Pathog..

[6] Park J., Lee B.-S., Choi J.-K., Means R.E., Choe J., Jung J.U. (2002). Herpesviral Protein Targets a Cellular WD Repeat Endosomal Protein to Downregulate T Lymphocyte Receptor Expression. Immunity.

[7] Fitzer-Attas C.J., Lowry M., Crowley M.T., Finn A.J., Meng F., Defranco A.L., Lowell C.A. (2000). Fcγ Receptor–Mediated Phagocytosis in Macrophages Lacking the Src Family Tyrosine Kinases Hck, Fgr, and Lyn. J. Exp. Med..

[8] Sicheri F., Moarefi I., Kuriyan J. (1997). Crystal structure of the Src family tyrosine kinase Hck. Nature.

[9] Xu W., Harrison S.C., Eck M.J. (1997). Three-dimensional structure of the tyrosine kinase c-Src. Nature.

[10] Yamaguchi H., Hendrickson W.A. (1996). Structural basis for activation of human lymphocyte kinase Lck upon tyrosine phosphorylation. Nature.

[11] Wiese N., Tsygankov A.Y., Klauenberg U., Bolen J.B., Fleischer B., Broker B.M. (1996). Selective activation of T cell kinase p56lck by Herpesvirus saimiri protein tip. J. Biol. Chem..

[12] Jung J.U., Lang S.M., Friedrich U., Jun T., Roberts T.M., Desrosiers R.C., Biesinger B. (1995). Identification of Lck-binding Elements in Tip of Herpesvirus Saimiri. J. Biol. Chem..

[13] Schweimer K., Hoffmann S., Bauer F., Friedrich U., Kardinal C., Feller S.M., Biesinger B., Sticht H. (2002). Structural investigation of the binding of a herpesviral protein to the SH3 domain of tyrosine kinase Lck. Biochemistry.

[14] Kim Y., Kwon E.K., Jeon J.H., So I., Kim I.G., Choi M.S., Kim I.S., Choi J.K., Jung J.U., Cho N.H. (2012). Activation of the STAT6 transcription factor in Jurkat T-cells by the herpesvirus saimiri Tip protein. J. Gen. Virol..

[15] Villarino A.V., Kanno Y., O’Shea J.J. (2017). Mechanisms and consequences of Jak-STAT signaling in the immune system. Nat. Immunol..

[16] May P., Gerhartz C., Heesel B., Welte T., Doppler W., Graeve L., Horn F., Heinrich P.C. (1996). Comparative study on the phosphotyrosine motifs of different cytokine receptors involved in STAT5 activation. FEBS Lett..

[17] Gaffen S.L., Lai S.Y., Xu W., Gouilleux F., Groner B., Goldsmith M.A., Greene W.C. (1995). Signaling through the interleukin 2 receptor beta chain activates a STAT-5-like DNA-binding activity. Proc. Natl. Acad. Sci. USA.

[18] Azam M., Erdjument-Bromage H., Kreider B.L., Xia M., Quelle F., Basu R., Saris C., Tempst P., Ihle J.N., Schindler C. (1995). Interleukin-3 signals through multiple isoforms of Stat5. EMBO J..

[19] Liu X., Robinson G.W., Gouilleux F., Groner B., Hennighausen L. (1995). Cloning and expression of Stat5 and an additional homologue (Stat5b) involved in prolactin signal transduction in mouse mammary tissue. Proc. Natl. Acad. Sci. USA.

[20] Chanda M.K., Shudde C.E., Piper T.L., Zheng Y., Courtney A.H. (2022). Combined analysis of T cell activation and T cell-mediated cytotoxicity by imaging cytometry. J. Immunol. Methods.

[21] Tyanova S., Temu T., Cox J. (2016). The MaxQuant computational platform for mass spectrometry-based shotgun proteomics. Nat. Protoc..

[22] Duboise S.M., Lee H., Guo J., Choi J.-K., Czajak S., Simon M., Desrosiers R.C., Jung J.U. (1998). Mutation of the Lck-Binding Motif of Tip Enhances Lymphoid Cell Activation by Herpesvirus Saimiri. J. Virol..

[23] Heck E., Friedrich U., Gack M.U., Lengenfelder D., Schmidt M., MüLler-Fleckenstein I., Fleckenstein B., Ensser A., Biesinger B. (2006). Growth Transformation of Human T Cells by Herpesvirus Saimiri Requires Multiple Tip-Lck Interaction Motifs. J. Virol..

[24] Wang K.S., Frank D.A., Ritz J. (2000). Interleukin-2 enhances the response of natural killer cells to interleukin-12 through up-regulation of the interleukin-12 receptor and STAT4. Blood.

[25] Gotthardt D., Putz E.M., Grundschober E., Prchal-Murphy M., Straka E., Kudweis P., Heller G., Bago-Horvath Z., Witalisz-Siepracka A., Cumaraswamy A.A. (2016). STAT5 Is a Key Regulator in NK Cells and Acts as a Molecular Switch from Tumor Surveillance to Tumor Promotion. Cancer Discov..

[26] Eckelhart E., Warsch W., Zebedin E., Simma O., Stoiber D., Kolbe T., Rülicke T., Mueller M., Casanova E., Sexl V. (2011). A novel Ncr1-Cre mouse reveals the essential role of STAT5 for NK-cell survival and development. Blood.

[27] Hedlund M., Nagaeva O., Kargl D., Baranov V., Mincheva-Nilsson L. (2011). Thermal- and Oxidative Stress Causes Enhanced Release of NKG2D Ligand-Bearing Immunosuppressive Exosomes in Leukemia/Lymphoma T and B Cells. PLoS ONE.

[28] Bryceson Y.T., March M.E., Ljunggren H.-G., Long E.O. (2006). Synergy among receptors on resting NK cells for the activation of natural cytotoxicity and cytokine secretion. Blood.

[29] Wang L., Zhang Y., Anderson E., Lamble A., Orentas R.J. (2022). Bryostatin Activates CAR T-Cell Antigen-Non-Specific Killing (CTAK), and CAR-T NK-Like Killing for Pre-B ALL, While Blocking Cytolysis of a Burkitt Lymphoma Cell Line. Front. Immunol..

[30] Navarrete-Galvan L., Guglielmo M., Cruz Amaya J., Smith-Gagen J., Lombardi V.C., Merica R., Hudig D. (2022). Optimizing NK-92 serial killers: Gamma irradiation, CD95/Fas-ligation, and NK or LAK attack limit cytotoxic efficacy. J. Transl. Med..

[31] Kirken R.A., Rui H., Malabarba M.G., Howard O.M., Kawamura M., O’Shea J.J., Farrar W.L. (1995). Activation of JAK3, but not JAK1, is critical for IL-2-induced proliferation and STAT5 recruitment by a COOH-terminal region of the IL-2 receptor beta-chain. Cytokine.

[32] Gorska M.M., Stafford S.J., Cen O., Sur S., Alam R. (2004). Unc119, a Novel Activator of Lck/Fyn, Is Essential for T Cell Activation. J. Exp. Med..

[33] Jang I.K., Zhang J., Chiang Y.J., Kole H.K., Cronshaw D.G., Zou Y., Gu H. (2010). Grb2 functions at the top of the T-cell antigen receptor–induced tyrosine kinase cascade to control thymic selection. Proc. Natl. Acad. Sci. USA.

[34] Lettau M., Pieper J., Janssen O. (2009). Nck adapter proteins: Functional versatility in T cells. Cell Commun. Signal..

[35] Bauer F., Schweimer K., Meiselbach H., Hoffmann S., Rösch P., Sticht H. (2005). Structural characterization of Lyn-SH3 domain in complex with a herpesviral protein reveals an extended recognition motif that enhances binding affinity. Protein Sci..

[36] Courtney A.H., Shvets A.A., Lu W., Griffante G., Mollenauer M., Horkova V., Lo W.L., Yu S., Stepanek O., Chakraborty A.K. (2019). CD45 functions as a signaling gatekeeper in T cells. Sci. Signal..

[37] Shimasaki N., Jain A., Campana D. (2020). NK cells for cancer immunotherapy. Nat. Rev. Drug Discov..

[38] Liu E., Marin D., Banerjee P., Macapinlac H.A., Thompson P., Basar R., Nassif Kerbauy L., Overman B., Thall P., Kaplan M. (2020). Use of CAR-Transduced Natural Killer Cells in CD19-Positive Lymphoid Tumors. N. Engl. J. Med..

[39] Cooley S., He F., Bachanova V., Vercellotti G.M., Defor T.E., Curtsinger J.M., Robertson P., Grzywacz B., Conlon K.C., Waldmann T.A. (2019). First-in-human trial of rhIL-15 and haploidentical natural killer cell therapy for advanced acute myeloid leukemia. Blood Adv..

[40] Miller J.S., Soignier Y., Panoskaltsis-Mortari A., McNearney S.A., Yun G.H., Fautsch S.K., McKenna D., Le C., Defor T.E., Burns L.J. (2005). Successful adoptive transfer and in vivo expansion of human haploidentical NK cells in patients with cancer. Blood.

[41] Bachanova V., Cooley S., Defor T.E., Verneris M.R., Zhang B., McKenna D.H., Curtsinger J., Panoskaltsis-Mortari A., Lewis D., Hippen K. (2014). Clearance of acute myeloid leukemia by haploidentical natural killer cells is improved using IL-2 diphtheria toxin fusion protein. Blood.

[42] Beatty G.L., O’Hara M.H., Lacey S.F., Torigian D.A., Nazimuddin F., Chen F., Kulikovskaya I.M., Soulen M.C., McGarvey M., Nelson A.M. (2018). Activity of Mesothelin-Specific Chimeric Antigen Receptor T Cells Against Pancreatic Carcinoma Metastases in a Phase 1 Trial. Gastroenterology.

[43] Barber-Rotenberg J.S., Haas A.R., Aggarwal C., O’Hara M., Hexner E., Pequignot E., Dowd E., Ndeupen S., Thai E., Chen F. (2026). Phase 1 study of autologous T cells bearing fully human chimeric antigen receptors targeting mesothelin in mesothelin-expressing cancers. Mol. Ther..

[44] Young K., Hughes D.J., Cunningham D., Starling N. (2018). Immunotherapy and pancreatic cancer: Unique challenges and potential opportunities. Ther. Adv. Med. Oncol..

[45] Imamura M., Shook D., Kamiya T., Shimasaki N., Chai S.M.H., Coustan-Smith E., Imai C., Campana D. (2014). Autonomous growth and increased cytotoxicity of natural killer cells expressing membrane-bound interleukin-15. Blood.

[46] Harris K.E., Lorentsen K.J., Malik-Chaudhry H.K., Loughlin K., Basappa H.M., Hartstein S., Ahmil G., Allen N.S., Avanzino B.C., Balasubramani A. (2021). A bispecific antibody agonist of the IL-2 heterodimeric receptor preferentially promotes in vivo expansion of CD8 and NK cells. Sci. Rep..

[47] Parkhurst M.R., Riley J.P., Dudley M.E., Rosenberg S.A. (2011). Adoptive Transfer of Autologous Natural Killer Cells Leads to High Levels of Circulating Natural Killer Cells but Does Not Mediate Tumor Regression. Clin. Cancer Res..

[48] Grange M., Buferne M., Verdeil G., Leserman L., Schmitt-Verhulst A.M., Auphan-Anezin N. (2012). Activated STAT5 promotes long-lived cytotoxic CD8^+^ T cells that induce regression of autochthonous melanoma. Cancer Res..

[49] Burchill M.A., Goetz C.A., Prlic M., O’Neil J.J., Harmon I.R., Bensinger S.J., Turka L.A., Brennan P., Jameson S.C., Farrar M.A. (2003). Distinct effects of STAT5 activation on CD4^+^ and CD8^+^ T cell homeostasis: Development of CD4^+^CD25^+^ regulatory T cells versus CD8^+^ memory T cells. J. Immunol..

[50] Ding Z.C., Shi H., Aboelella N.S., Fesenkova K., Park E.J., Liu Z., Pei L., Li J., McIndoe R.A., Xu H. (2020). Persistent STAT5 activation reprograms the epigenetic landscape in CD4^+^ T cells to drive polyfunctionality and antitumor immunity. Sci. Immunol..

[51] Gasser S., Corthesy P., Beerman F., MacDonald H.R., Nabholz M. (2000). Constitutive expression of a chimeric receptor that delivers IL-2/IL-15 signals allows antigen-independent proliferation of CD8^+^CD44^high^ but not other T cells. J. Immunol..

[52] Sockolosky J.T., Trotta E., Parisi G., Picton L., Su L.L., Le A.C., Chhabra A., Silveria S.L., George B.M., King I.C. (2018). Selective targeting of engineered T cells using orthogonal IL-2 cytokine-receptor complexes. Science.

[53] Cho W., Liu J.Y., Beckett A.N., Lunger J.C., Xu P., Ho K., Chen E.E., Salcido-Alcántar A., Sant’Anna L.E., Obbad K. (2025). A continuous landscape of signaling encodes a corresponding landscape of CAR T cell phenotype. bioRxiv.

[54] Di Stasi A., Tey S.K., Dotti G., Fujita Y., Kennedy-Nasser A., Martinez C., Straathof K., Liu E., Durett A.G., Grilley B. (2011). Inducible apoptosis as a safety switch for adoptive cell therapy. N. Engl. J. Med..

[55] Straathof K.C., Pule M.A., Yotnda P., Dotti G., Vanin E.F., Brenner M.K., Heslop H.E., Spencer D.M., Rooney C.M. (2005). An inducible caspase 9 safety switch for T-cell therapy. Blood.

