# Supplementary material for: Characterization of a Cytokine-Independent STAT5 Activator
Source: Biomedicines. 2026 May 13;14(5):1097. doi: 10.3390/biomedicines14051097 (PMC13204663; doi:10.3390/biomedicines14051097)
Supplement: Supplementary file 1 [file biomedicines-14-01097-s001.zip › Aleck et al Biomedicines 04_2026_SI_revised_FINAL.pdf]

SUPPLEMENTAL INFORMATION

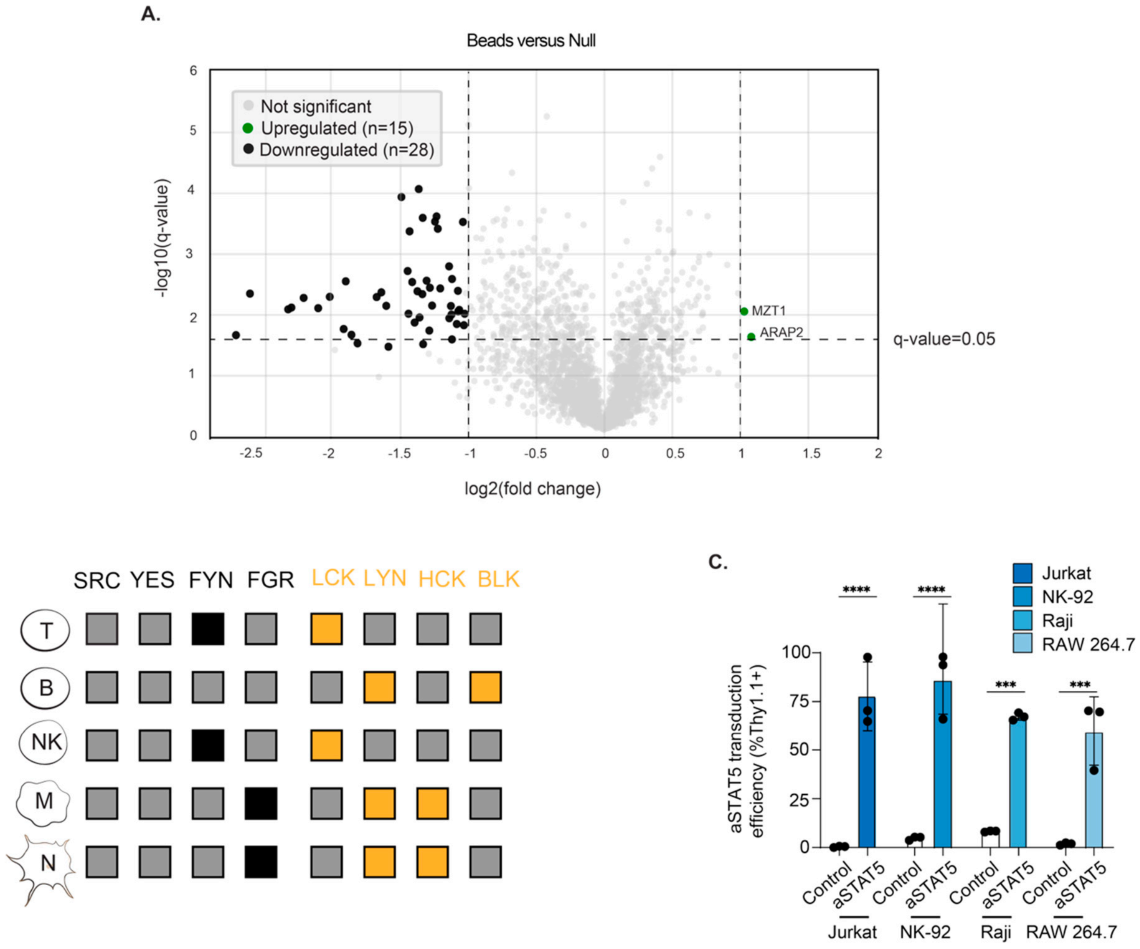

Figure S1. Additional comparisons of AP-MS controls and transduction efficiency of cell lines. (**A**) Volcano plot of differentially enriched proteins between Beads and Null highlighting ($q < 0.05$, log2FC > 1.0) in green. Black denotes reduced proteins ($q < 0.05$, log2FC < -1.0). (**B**) SFK family members found in hematopoietic cells. (**C**) A panel of hematopoietic cell lines was transduced with aSTAT5 containing a Thy1.1 marker or a control plasmid. Transduction efficiency was assessed by flow cytometry and percentage of Thy1.1 positive cells was calculated. (n = 3, means ± SD); ***$P < 0.001$; ****$P < 0.0001$; two-way ANOVA, Šidák's multiple comparisons.

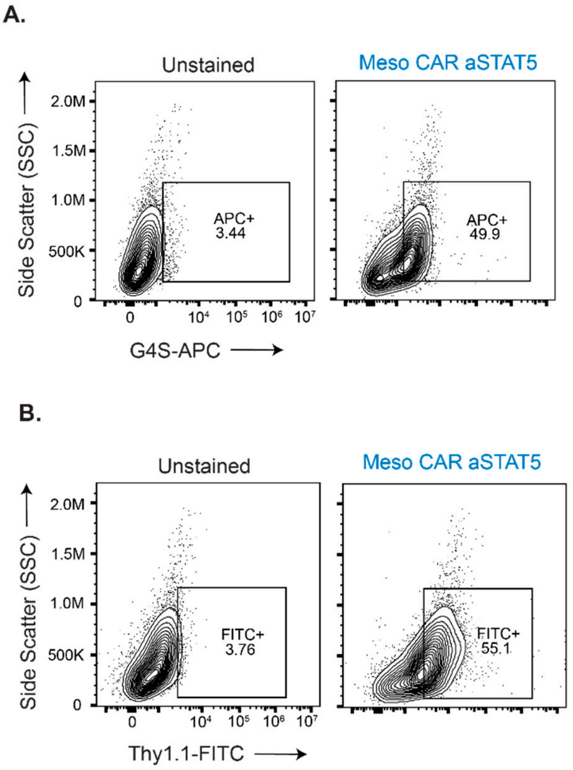

Figure S2. Human CD8+ T cells transduced to express a mesothelin (MSLN)-specific CAR and aSTAT5. (**A**) A mesothelin-specific chimeric antigen receptor (CAR) and (**B**) aSTAT5 were stably transduced into human CD8+ T cells using lentivirus. The percentage of the cell population expressing the CAR and aSTAT5 is annotated above. These values were assessed using flow cytometry with antibody staining for the Thy1.1 marker in the aSTAT5 containing plasmid and the poly-Glycine-Serine (G4S) linker contained in the MSLN-specific CAR. Gates were drawn using an unstained human CD8+ T cell population as a negative control.

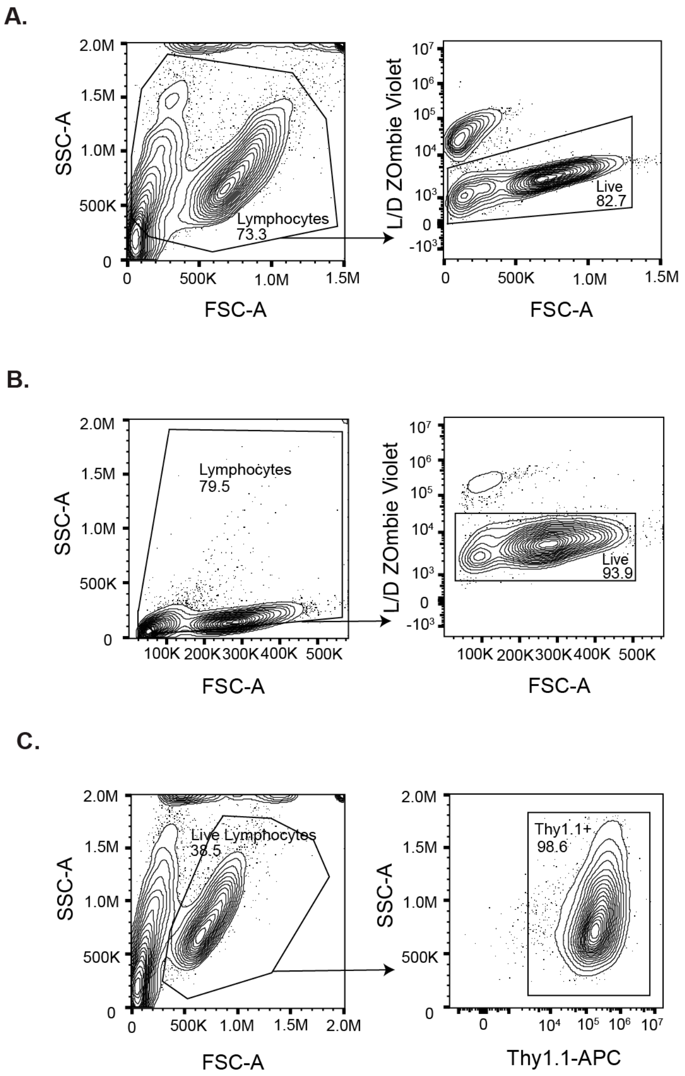

Figure S3. Quantification of cell viability and transduction efficacy. (**A**) Representative gating strategy used to assess population of live NK-92 cells from total lymphocyte population, (**B**) live human CD8+ T cells from total lymphocyte population, and (**C**) live transduced NK-92 cells from a live lymphocyte population identified by the expression of Thy1.1 as a marker contained in the pHR EF1a aSTAT5 IRES Thy1.1 plasmid. The percentages of the annotated cell populations are included below the identified population.

Antibody	Vendor	Catalogue Number	Dilution	MW (kDa)
CD90.1 (Thy-1.1) APC (OX-7)	Biolegend	202526	1:200	n/a
CD90.1 (Thy-1.1) FITC (OX-7)	Biolegend	202503	1:200	n/a
G4S Linker (E702V) APC	Cell Signaling Technology	68718S	1:200	n/a
SFK pY416 (LCK pY394)	Cell Signaling Technology	2101	1:2000	60
GAPDH (D16H11)	Cell Signaling Technology	5174	1:2000	37
MYC Tag (9B11)	Cell Signaling Technology	2276	1:1000	n/a
STAT5 pY694 (D47E7)	Cell Signaling Technology	4322	1:2000	90
STAT5 (D206Y)	Cell Signaling Technology	94205	1:2000	90
Goat anti-mouse IgG(H+L), Human ads-HRP	Southern Biotech	1031-05	1:5000	n/a
Goat anti-rabbit IgG(H+L), Human ads-HRP	Southern Biotech	4050-05	1:5000	n/a
Anti-c-Myc Magnetic Beads	ThermoFischer	88842	n/a	n/a
Ultra-LEAF Purified anti-human CD3 (OKT3)	Biolegend	317326	1:200	n/a
Ultra-LEAF Purified anti-human CD28 (CD28.2)	Biolegend	302934	1:400	n/a

Table S1. Antibodies used.

Accession	Gene Symbol	log2FC	pvalue	qvalue
P42229	STAT5A	2.462823	0.000931	0.005641
Q8WZ64	ARAP2	1.514551	0.003163	0.013074
O75431	MTX2	1.470797	0.003224	0.012721
P01730	CD4	1.340134	0.007403	0.019302
Q13432	UNC119	1.190118	0.0071	0.020158
Q9Y421	FAM32A	1.11934	0.012093	0.030484
Q16891	IMMT	1.117979	0.007536	0.019355
Q9BRQ6	CHCHD6	1.097514	0.007159	0.020059
Q96HY6	DDRGK1	1.076553	0.001525	0.009467
Q9NX63	CHCHD3	1.074793	0.00171	0.008353
Q6NZ67	MZT2B	1.07391	0.011079	0.027022
P09622	DLD	1.071142	0.000958	0.0055
Q08722	CD47	1.028893	0.000423	0.0062

Table S2. Top enriched proteins by log2FC out of all proteins identified in aSTAT5 vs. Minimal.

Accession	Gene Symbol	log2FC	pvalue	qvalue
P19174	PLCG1	0.929214	2.124102	0.019033
P06239	LCK	0.842867	3.138507	0.007096
P41240	CSK	0.016269	0.047053	0.948858
P16333	NCK1	-1.200917	1.947311	0.026964
P40763	STAT3	0.1703333	0.329225	0.597510
P42224	STAT1	0.045156	0.293491	0.639808

Table S3. Additional proteins of interest out of all proteins identified in aSTAT5 vs. Minimal, selected annotated in Fig. 2B.

Kinase	Immune Cell Type
LCK	T cells, NK cells
FYN	T cells, NK cells
LYN	B cells, myeloid cells
BLK	B cells
HCK	Monocytes, macrophages, neutrophils
FGR	Monocytes, neutrophils

Table S4. Src family kinases expressed in immune cells.
